# FPGA-Based Particle Swarm Collaborative Target Localization Algorithm for UAV Swarms

**DOI:** 10.3390/s25082462

**Published:** 2025-04-14

**Authors:** Chuanhao Zhang, Changsheng Li, Zhipeng Chen, Haojie Li, Hang Yu

**Affiliations:** School of Mechanical Engineering, Nanjing University of Science and Technology, Nanjing 210094, China; chuanhao.zhang@njust.edu.cn (C.Z.); lichangsheng1984@163.com (C.L.); czp@njust.edu.cn (Z.C.)

**Keywords:** cooperative target localization, particle swarm optimization algorithm, FPGA deployment and verification, algorithm localization capability analysis

## Abstract

To achieve precise collaborative localization of multiple unmanned aerial vehicles (UAVs) in hardware environments, this paper presents an field-programmable gate array-based particle swarm optimization (PSO) algorithm aimed at enhancing the localization efficiency of multiple nodes targeting a specific object. By leveraging the unique computational capabilities of FPGA, the proposed algorithm integrates optimization strategies, including particle mutation, variable crossover probabilities, and adjustable weights. These strategies collectively enhance the performance of the PSO algorithm in localization tasks. Comparative simulations conducted across a range of operational scenarios demonstrate that the algorithm not only ensures high localization accuracy but also delivers excellent real-time performance and rapid convergence. To further validate the algorithm’s practical applicability, a four-node collaborative localization platform was developed, and experiments were carried out. The results confirmed the feasibility of multi-node collaborative localization, underscoring the advantages of the proposed algorithm, such as high accuracy, fast convergence, and robust stability.

## 1. Introduction

A single node can carry either a single or a composite sensor for precise target ranging but cannot achieve accurate target positioning. The method proposed in [1] relies on a single node for target localization, but due to the limited numbr of nodes, the positioning accuracy remains low. However, multiple UAVs, utilizing wireless communication technology, can share detection information among multiple UAVs, thus enabling the determination of the target’s three-dimensional coordinates.

With the rapid advancement of wireless sensor network technology, multi-point collaborative localization has been widely applied in areas such as military operations, environmental monitoring, intelligent transportation, and underwater surveillance. In field emergency rescue and search-and-rescue operations, multiple UAVs can collaboratively localize the positions of individuals in distress at accident sites or disaster areas, thereby enhancing search efficiency. During infrastructure inspection and security monitoring. For example, when inspecting large structures such as bridges, highways, or wind farms, multiple UAVs can accurately pinpoint areas of damage. Additionally, in the event of traffic accidents, precise localization of the involved vehicles or tracking of the offending vehicles is possible. Therefore, designing high-performance localization algorithms and implementing them in hardware can provide critical technical support for engineering applications involving the collaborative localization of unknown passive targets by multiple UAVs.

Common multi-point positioning methods include trilateration and angle-based positioning. Trilateration allows for the direct or indirect measurement of parameters such as signal transmission time and strength between the measurement nodes and the target. Techniques such as time difference of arrival (TDOA) [2], time of arrival (TOA) [3,4], time of flight (TOF) [5], and received signal strength indicator (RSSI) [6] are employed to establish nonlinear equations for solving the target’s position. Angle-based positioning, which uses methods like angle of arrival (AOA) [7], determines the pitch and azimuth angles between the measurement nodes and the target, subsequently forming nonlinear equations to compute the target’s location. Common methods to solve these equations include Newton’s method [8], the Chan method [9], and the least squares method [10]. However, these methods have their drawbacks. Newton’s method is sensitive to initial values and may fail to converge if they are far from the correct solution; the Chan method struggles with measurement noise and nonlinear problems; and the least squares method, though accurate, requires complex matrix inversion, making it impractical for weak hardware environments. Swarm intelligence algorithms [11], commonly used for single or multi-objective optimization, outperform traditional methods, offering better convergence for both linear and nonlinear problems. By establishing an appropriate objective function, the positioning task can be transformed into a minimization problem. Currently, swarm intelligence algorithms are frequently employed to address the localization challenges of unknown nodes in wireless sensor networks.

Although swarm intelligence algorithms speed up problem-solving, they are prone to getting stuck in local optima during iterations. As a result, most researchers optimize and simulate these algorithms on high-level software platforms. The paper [12] introduced dynamic self-adaptive tournament-based genetic algorithm (dynsTGA) and hybrid evolutionary heuristic optimization (HEHO) algorithms and analyzed the impact of measurement noise on positioning accuracy through software simulations, validating the effectiveness of the optimized algorithms. The paper [13] presented a rat swarm optimization algorithm and, by adding Gaussian white noise to the measurement data, compared the algorithm with others using MATLAB R2022a simulations, demonstrating a 60 percent reduction in positioning error. The paper [14] designed a cuckoo optimization algorithm and used software simulations to assess its performance under the assumption of no node positioning errors, achieving a target positioning error of 0.259 m. The paper [15] proposed an optimized bat algorithm, investigating the effects of ranging errors and the number of positioning nodes on ranging errors and solution time through the top-level software simulations. Although the original bat algorithm offered high accuracy, its solution time was long. The optimized algorithm, inspired by the bacterial foraging algorithm’s chemotactic motion, significantly improved convergence speed. In paper [16], a hybrid algorithm of butterfly optimization algorithm (BOA) and PSO algorithm is proposed, which incorporates the chaos theory into the inertia weight parameter of PSO algorithm and adopts an adaptive strategy to update the value of the sensory fragrance of BOA, and the optimized algorithm’s localization performance is improved as proved by the function test of CEC2014. Similarly, the paper [17] improves the search capability of the algorithm by adaptively adjusting parameters such as inertia weights, learning factor. The paper [18] combined bacterial foraging optimization (BFO) and PSO algorithms for optimization to avoid local optima. Similarly, the paper [19] combined grey wolf optimization (GWO) and PSO algorithms for optimization. While these optimized algorithms showed good performance in simulations, their effectiveness in hardware environments remains to be proven. The paper [20] improved the genetic algorithm and simulated it on a software platform, verifying its ability to maintain high positioning accuracy even under significant measurement error variance. The paper [21] addressed the ranging errors caused by multi-hop information transmission and conducted simulation studies, though it lacked a quantitative analysis of positioning errors and various error types.

FPGA is a highly flexible integrated circuit endowed with parallel processing capabilities. It is composed of numerous independent logic blocks and interconnection resources, enabling it to execute multiple computational tasks simultaneously. Because it employs dedicated hardware logic for task processing, FPGA exhibits low latency in data transmission and computation, thereby meeting the demands of applications requiring high real-time performance. Consequently, integrating the collaborative positioning algorithm with FPGA enables rapid solution convergence.

Most of the aforementioned research is based on top-level software platforms, focusing on target positioning in two-dimensional coordinate systems. These algorithm solutions typically rely on high-precision floating-point operations and built-in software functions for complex calculations. While these approaches verify the effectiveness of algorithm optimization, they do not explore the actual performance of the algorithm in hardware environments with limited resources. Additionally, some studies fail to quantitatively analyze the impact of measurement errors on positioning accuracy, making it difficult to assess the true capability of the algorithms. In target collaborative detection, high positioning accuracy is essential, but response speed is equally crucial. Although software simulations can verify the theoretical feasibility of optimization algorithms, they cannot accurately predict the precision, speed, and stability in hardware environments.

Therefore, this paper optimizes the particle swarm algorithm based on the FPGA’s logical structure, with ModelSim serving as the simulation platform for hardware operation. This approach improves the algorithm’s performance in practical applications. By utilizing this hardware simulation platform, the impact of ranging errors on positioning performance is thoroughly considered. Various operating conditions are simulated and compared to verify the algorithm’s reliability and speed. Additionally, a multi-node collaborative positioning module is constructed, and experimental verification is conducted to demonstrate the feasibility of the optimized algorithm and its advantages in terms of positioning accuracy, computation time, and other performance metrics. The specific work done and the main contributions are as follows:

1. Most researchers design swarm intelligence optimization positioning algorithms using high-level development environments, which do not guarantee positioning accuracy in hardware with limited resources. This paper proposes a modular design for the particle swarm positioning algorithm based on FPGA, enabling hardware deployment and simulation analysis of the algorithm.
2. To address the challenge of local optima in the particle swarm positioning algorithm when implemented on FPGA in a finite-width fixed-point environment, this work integrates ideas from differential evolution and simulated annealing algorithms. By incorporating variable crossover probabilities and adjustable weights, the proposed method optimizes the performance of the particle swarm positioning algorithm.
3. While most studies [22,23,24] focus on comparative simulations, making it difficult to assess algorithm performance in real-world conditions, this paper overcomes this limitation by constructing a four-node collaborative positioning platform. Through a distributed communication structure, multi-node collaborative positioning is achieved. The algorithm’s positioning accuracy, stability, and solving speed are verified under practical conditions, providing valuable technical support for the application of collaborative target positioning algorithms.

Section 2 presents the design, optimization, and FPGA deployment process of the particle swarm cooperative localization algorithm. Section 3 details a comparative simulation analysis between the proposed VCA-PSO algorithm and other algorithms, and examines the relationship between different ranging errors and localization errors. Section 4 describes the collaborative localization experiment, demonstrating the feasibility, speed, and accuracy of the collaborative localization algorithm developed in this paper. Section 5 provides the main conclusions derived from this study.

## 2. The Design and Optimization of FPGA-Based Particle Swarm Cooperative Localization Algorithm

### 2.1. Collaborative Localization Model Based on Particle Swarm Algorithm

In emergency rescue scenarios at sea, such as sinking vessels or individuals in the water, multiple UAVs can be deployed to remotely survey the incident area. However, since targets like sunken ships do not actively provide location information, only the distances between the target area and the detection nodes can be obtained remotely using sensors such as infrared, laser, and ultrasonic devices. The ranging data are then transmitted to a central node via a distributed communication network. The central node uses this data, in conjunction with the known positions of the UAV nodes, to solve an objective function and predict the exact location of the target. Consequently, rescue vessels or helicopters can be directed to the predicted location to carry out the rescue operation.

For this multi-node collaborative localization scenario, the target localization problem is formulated as a nonlinear optimization problem.

The PSO algorithm [25] is an evolutionary computation method inspired by the flocking behavior of birds and schooling of fish. In PSO, each solution—referred to as a particle—is defined by its position vector $X=(X_{1},X_{2},\ldots ,X_{n})$ and its velocity $V=(V_{1},V_{2},\ldots ,V_{n})$. For example, a particle might be represented as $X_{1}=\left(x_{1},y_{1},z_{1}\right)$ and $V_{1}=\left(v_{x1},v_{y1},v_{z1}\right)$. Each particle iteratively adjusts its position by considering its own best historical position, $P_{g}$, and the best position found by the entire swarm, $G_{g}$. This adjustment is achieved by updating the velocity according to the following Equation (1).

(1) $$V_{\left(g+1\right)}=\omega V_{g}+c_{1}rand\left(P_{g}-X_{g}\right)+c_{2}rand\left(G_{g}-X_{g}\right)$$

where ω is the inertia factor; *g* represents the *g*-th iteration; $c_{1}$, $c_{2}$ are the learning factors; rand is the random number between 0 and 1; $P_{g}$, $G_{g}$ are the individual optimal position and the global optimal position, respectively.

The particle completes the position update through Equation (2).

(2) $$X_{\left(g+1\right)}=X_{g}+V_{g}$$

A crucial component of PSO is the fitness evaluation, which determines the ‘goodness’ of each particle. In the application of joint target localization using multiple sensor nodes, the target is denoted by T=(x,y,z). To estimate the target location, a fitness function F(X) is defined based on the discrepancies between the measured distances and the distances computed from a candidate position. Suppose each sensor node *i* is located at $\left(x_{si},y_{si},z_{si}\right)$ and measures a distance $d_{i}$ to the target. The fitness function is then formulated the following Equation (3).

(3) $$F\left(X\right)=\sum_{i=1}^{n}|\sqrt{{\left(x-x_{si}\right)}^{2}+{\left(y-y_{si}\right)}^{2}+{\left(z-z_{si}\right)}^{2}}-d_{i}|$$

Ideally, the optimal target location $X_{p}=\left(x_{p},y_{p},z_{p}\right)$ would satisfy $F(X_{p})=0$. However, due to measurement uncertainties, the goal is to minimize F(X) so that the predicted target position is as close as possible to the true location. In this way, the localization challenge is transformed into an optimization problem focused on finding the minimum of the fitness function.

### 2.2. FPGA Deployment of Particle Swarm Localization Algorithm

**Figure 1:**
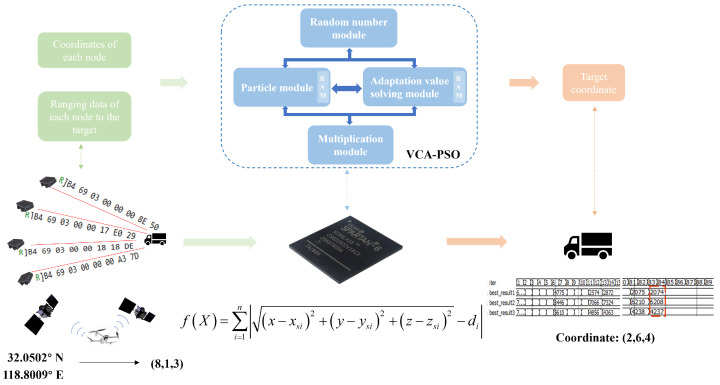
Schematic diagram of FPGA solving target coordinates.

To implement the collaborative positioning algorithm, PSO based positioning algorithm is deployed on FPGA. By leveraging the node positions and ranging information, the main node performs the cooperative, precise localization of the target. The overall process is illustrated in Figure 1.

Where the red dashed box is the target position obtained by the algorithm solution.

**Figure 2:**
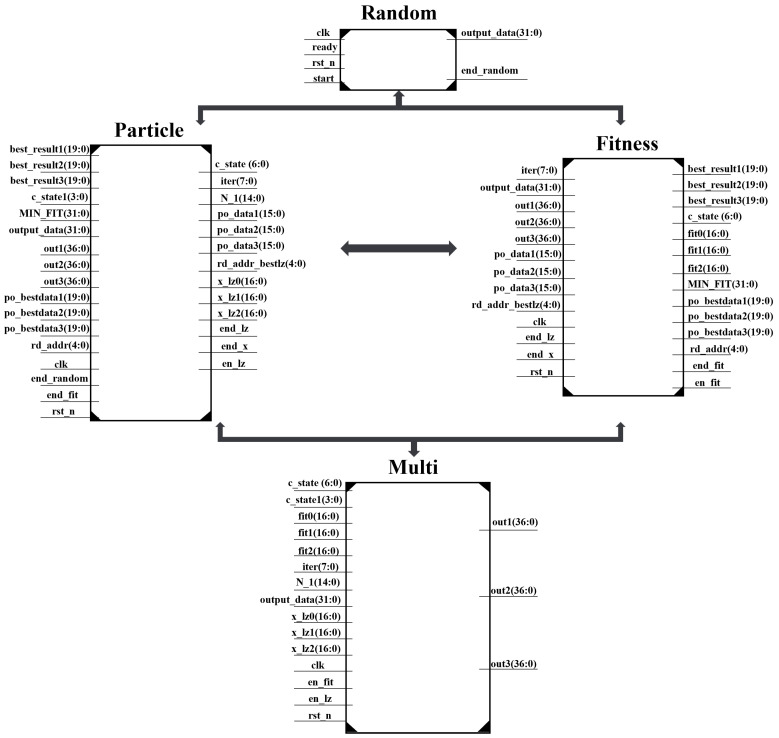
Module input/output relationships and ports.

We designed several modules to handle different steps of the algorithm as Figure 2 shows. These modules ensure reliable algorithm execution while accelerating the iteration speed through parallel computation, enabling quick and accurate determination of the target position.

Four key modules were designed based on the algorithm-solving flow: the Random Number Module (Random), Particle Velocity and Position Update Module (Particle), Fitness Comparison Module (Fitness), and Multiplication Computation Module (Multi). Each module is controlled by a state machine to execute operations such as fitness calculation, particle update, data reading, and storage. The specific functions of each module are as follows:

Random Number Module: The random number module is responsible for generating the random numbers required by the algorithm, including the initial positions and velocities of the particles, as well as the random numbers needed for velocity updates. Unlike software platforms, FPGA logic units typically perform calculations using integers and are less sensitive to decimal computations. Therefore, high-quality randomness is essential for initializing particles and calculating the fitness function. While most studies [26,27,28] typically use the Linear Feedback Shift Registers (LFSR) method to generate random numbers, this paper opts to utilize the Mersenne Twister random number generator. The Mersenne Twister produces random numbers via a linear recursion and offers superior statistical properties. It generates uniformly distributed random sequences with lower correlations between successive random numbers.

Particle Module: The Particle Module primarily handles the initialization of the particles’ velocity and position information. It also updates the particle positions and velocities at each iteration. Additionally, this module checks the boundaries of the updated velocities and positions. This module contains six dual-port RAMs that store the updated particle positions and velocities.

Fitness Comparison Module: The Fitness Comparison Module computes the fitness values of the particles at each iteration. This module compares the fitness of each particle to identify the individual best solution and extracts the global best solution from this. This module contains four dual-port RAMs to store each particle’s individual best solution and the corresponding optimal fitness values for each of the three coordinate axes.

Multiplication Computation Module: The Multiplication Computation Module performs multiplication calculations for various terms in the objective function and velocity update equations. This module primarily utilizes FPGA’s DSP48A1 resources to execute high-speed calculations.

The pseudocode for PSO positioning method based FPGA is as Algorithm 1.
**Algorithm 1:** PSO positioning algorithm
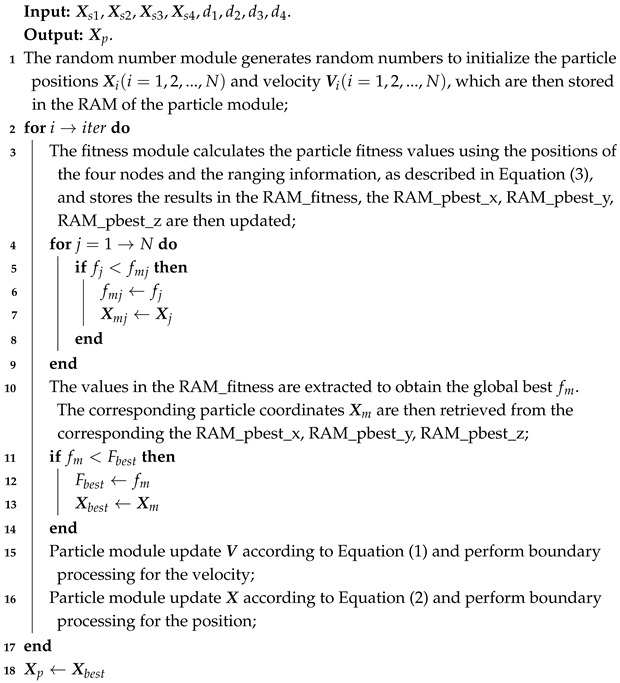


Where $X_{s1},X_{s2},X_{s3},X_{s4}$ are the coordinates of the four detected nodes. $d_{1},d_{2},d_{3},d_{4}$ are the respective detected distance information. $X_{p}$ is the target position obtained by solving the algorithm. iter is the maximum number of iterations. $f_{j}$ is the fitness values of each particle during each iteration. $f_{mj}$ is the historical minimum individual fitness value of a particle. $X_{j}$ is the coordinate of a particle. $X_{mj}$ is the coordinate correspond to $f_{mj}$. $F_{best}$ is the historical minimum fitness of all particles. $X_{best}$ is the coordinate correspond to $F_{best}$.

### 2.3. Optimized Localization Algorithm—VCA-PSO

Using multi-bit fixed-point numbers for algorithm computations on FPGA would consume a large number of register resources and increase hardware power consumption. Therefore, under the constraints of limited bit width, reducing the inherent errors of PSO algorithm and the computational errors of the FPGA is a key focus of this study. To address this, this paper combines the mutation and crossover operations of DE algorithm with the principles of SA algorithm and designs a strategy for dynamically adjusting the crossover probability. This strategy ensures that the algorithm maintains a broad search space in the early iterations, progressively reducing randomness as the algorithm proceeds.

Mutation Operation: The mutation operation is performed on selected particles to generate new solutions. The process is described by Equation (4).

(4) $$M_{\left(g+1\right)}=r_{i}\left(g\right)+F\left[\left({\mathrm{pbest}}_{r_{1}\left(g\right)}-r_{i}\left(g\right)\right)+\left({\mathrm{pbest}}_{r_{2}\left(g\right)}-{\mathrm{pbest}}_{r_{3}\left(g\right)}\right)\right]$$

where *g* represents the current number of iterations; $r_{i}\left(g\right)$ is the selected particle; pbest represents the individual best solutions of particles $r_{1},r_{2},r_{3}$, which are randomly selected in the population and satisfy $r_{1}\neq r_{2}\neq r_{3}\neq i$; *M* represents the position solution after mutation. *F* signifies the mutation factor. The scaling factor *F* determines the magnitude of the mutation of the particle.

Crossover Operation: The crossover operation involves combining the mutated solution with the original solution to generate a new solution.

(5) $$U_{i(g+1)}=\left\{\begin{array}{c} M_{i,j}\left(g+1\right),\;if\;rand\left(0,1\right)\leq CR\\ X_{i,j}\left(g\right),\;otherwise \end{array}\right.$$

The crossover probability, denoted by CR, controls which particles will participate in the crossover operation. In this context, $X_{i,j}$ represents the *j*-th coordinate of the *i*-th particle, $U_{i(g+1)}$ represents the *j*-th coordinate of the new particle, $M_{i,j}\left(g+1\right)$ represents the *j*-th coordinate of the mutated particle, and rand is a random number between 0 and 1.

In traditional DE algorithm, the crossover probability CR is a constant. However, in FPGA implementation, the crossover probability should gradually decrease as the algorithm progresses, to ensure better convergence in the later stages. The core idea of the SA algorithm is to introduce a temperature parameter that probabilistically accepts worse solutions to avoid local optima. We adopt this principle and design a crossover probability function such that CR remains high during the initial stages to explore the solution space fully, and gradually decreases as the iterations proceed to enhance convergence. To achieve this, we have designed a crossover probability function that decreases with increasing iteration number, represented by Equation (6).

(6) $$CR=\left\{\begin{array}{c} 0.9-0.4e^{0.1(iter-k_{1})},\;if\;1\leq iter\leq k_{1}\\ 0.5,\;if\;k_{1}<iter\leq iter_{max} \end{array}\right.$$

In the PSO algorithm, the inertia weight ω determines the balance between global and local search capabilities of the particles. A larger inertia weight helps the particles perform global search, while a smaller weight enhances local search ability. To optimize the search process, this paper proposes a dynamically adjustable inertia weight formula, as shown in Equation (7).

(7) $$\omega =\left\{\begin{array}{c} \omega_{min}+\left(\omega_{max}-\omega_{min}\right)e^{-\alpha \frac{iter}{iter_{max}}}+\sigma \cdot normrnd\left(p,q\right),\;if\;1\leq iter\leq k_{2}\\ \omega_{min}+\left(\omega_{max}-\omega_{min}\right)e^{-\alpha \frac{k_{2}}{iter_{max}}}+\sigma \cdot normrnd\left(p,q\right),\;if\;k_{2}<iter\leq iter_{max} \end{array}\right.$$

where $\omega_{min}$ and $\omega_{max}$ represent the minimum and maximum values of the inertia weight, respectively, iter is the current iteration number, $iter_{max}$ is the maximum iteration count, σ is a random correction factor, and α is the decay factor. For ease of FPGA computation, we linearized the formulas for both the crossover probability and inertia weight, leading to a simplified formula that balances global and local search demands throughout the search process.

The pseudocode for VCA-PSO positioning method based FPGA is as Algorithm 2.
**Algorithm 2:** VCA-PSO positioning algorithm
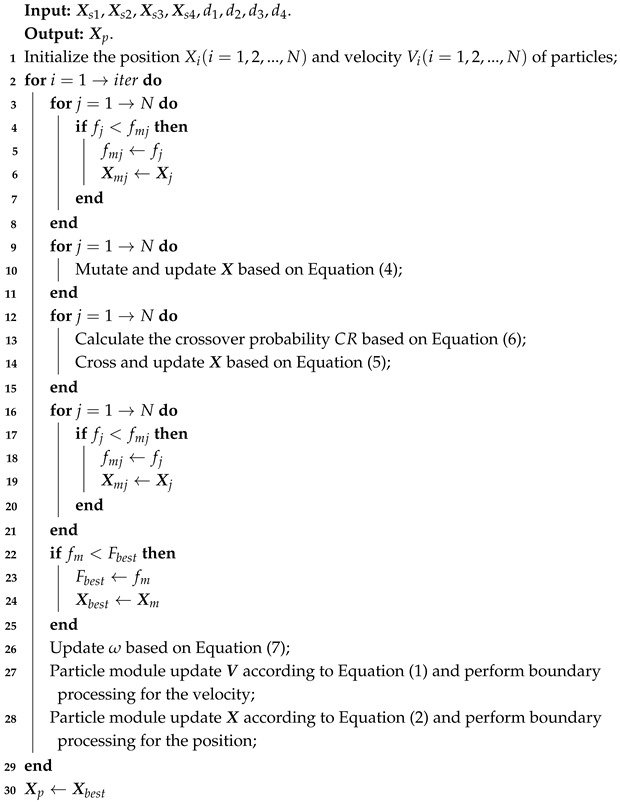


Where $X_{s1},X_{s2},X_{s3},X_{s4}$ are the coordinates of the four detected nodes. $d_{1},d_{2},d_{3},d_{4}$ are the respective detected distance information. $X_{p}$ is the target position obtained by solving the algorithm. iter is the maximum number of iterations. $f_{j}$ is the fitness values of each particle during each iteration. $f_{mj}$ is the historical minimum individual fitness value of a particle. $X_{j}$ is the coordinate of a particle. $X_{mj}$ is the coordinate correspond to $f_{mj}$. $F_{best}$ is the historical minimum fitness of all particles. $X_{best}$ is the coordinate correspond to $F_{best}$.

### 2.4. VCA-PSO Algorithm Time Complexity Analysis

Given that the algorithm iterates for a total of iter times, the overall time complexity can be derived by multiplying the time complexity per iteration by the number of iterations.

Assuming there are *N* particles, the time complexity for each evaluation of the fitness function in the main loop is O(N·F). The time complexities for the particle mutation and variable probability crossover operations are O(N·d), and similarly, the time complexity for updating the velocity and position is O(N·d). Scalar updates and comparisons incur a time complexity of O(1). Therefore, the time complexity per iteration is O(N·(F+d)). Consequently, with iter iterations, the overall time complexity of the algorithm is O(iter·N·(F+d)).

## 3. Simulation Verification of FPGA-Based Localization Algorithm

ModelSim is a simulation and debugging platform for hardware description languages (HDLs) that is widely used in digital circuit design and verification. It supports various HDLs, including VHDL, Verilog, and SystemVerilog, and can accommodate a range of design requirements. ModelSim facilitates rapid simulation of large-scale designs on FPGA, thereby shortening the verification cycle. In FPGA projects, ModelSim is utilized for early-stage functional verification and debugging to ensure that the design meets its intended performance before hardware implementation.

### 3.1. Simulation Verification of FPGA-Based PSO Localization Algorithm

**Figure 3:**
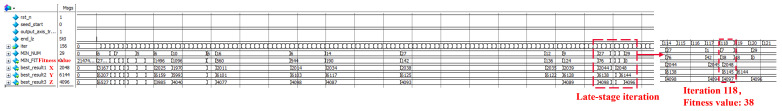
Simulating the iterative process of running the PSO positioning algorithm on FPGA.

Behavioral simulation on the ModelSim platform can simulate the actual operation of the algorithm on FPGA to evaluate the feasibility and timing performance of the hardware implementation. The simulation is set with a clock frequency of 50 MHz, a time precision of 1 ps, a fixed-point width of 10 bits, 30 particles, and a maximum iteration count of 200. The target position is set to (2, 6, 4), with input parameters being the positions of the four nodes: (8, 2, 7), (10, 3, 1), (9, 2, 2), and (8, 1, 3), along with the Euclidean distances from each node to the target. The default ranging error is set to 0. The FPGA simulation of the PSO algorithm iteration process is shown in Figure 3.

In Figure 3, iter represents the iteration count; MIN_NUM is the global best particle index; MIN_FIT is the minimum fitness value; Best_result1, Best_result2, and Best_result3 represent the *x*, *y*, and *z* coordinates of the best particle, respectively. The simulation results show that as the number of iterations increases, the MIN_FIT gradually decreases, and the particles progressively converge towards the target position. By around the 120th iteration, the fitness value reaches 0, and the FPGA-derived global best solution is (2048, 6144, 4096), which is highly consistent with the actual target coordinates (2, 6, 4), proving the feasibility of the FPGA-based PSO localization algorithm.

**Figure 4:**
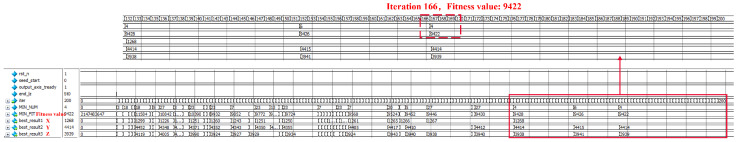
The case of falling into a local optimum in solving for objective coordinates.

PSO algorithms are prone to local optima during the early iterations if the global search ability is insufficient, which can result in increased localization errors. FPGA-based calculations using fixed-point arithmetic with limited bit-width can introduce inherent quantization errors, increasing the likelihood of the algorithm becoming stuck in local optima. Therefore, simulating the localization algorithm on a hardware platform provides results closer to practical conditions. The simulation result for the case of falling into a local optimum is shown in Figure 4.

In the simulation process, the initial fitness value was 11,584. As the iterations progressed, the fitness value (MIN_FIT) did not decrease significantly. After 166 iterations, the fitness value remained at 9422, with the computed target coordinates being (1268, 4414, 3939), corresponding to a localization error of 1.8 m. This discrepancy arises from the differences in working mechanisms between top-level software and hardware architecture.

**Figure 5:**
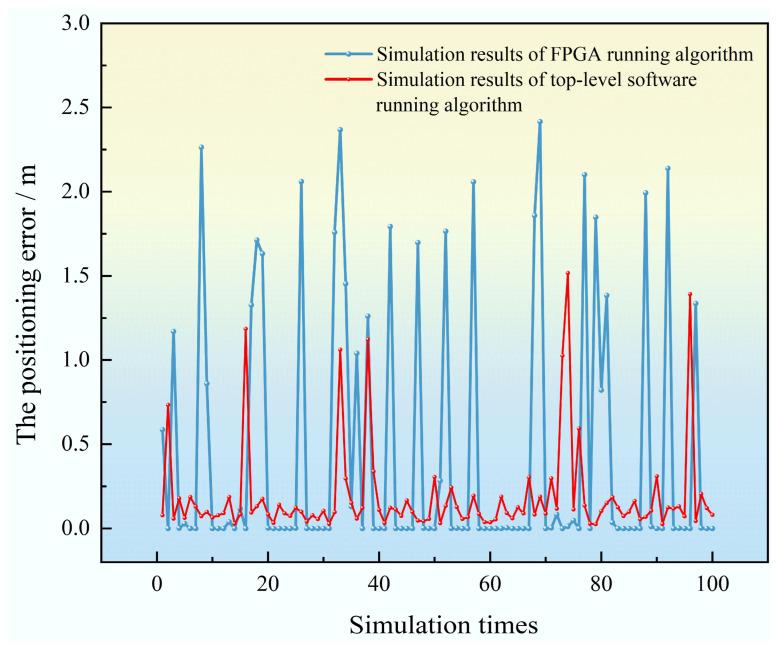
Positioning error of simulated FPGA running the positioning algorithm 100 times.

To further evaluate the performance of the designed localization algorithm, 100 simulations were run based on the FPGA computation architecture. The comparison of simulation results is shown in Figure 5.

From the 100 simulations, the average localization error of the PSO algorithm running on FPGA was 0.4351 m, while the average error from the top-level software simulation was 0.209 m. The results show that in the 100 simulations conducted on the top-level software, cases where the localization error exceeded 1 m occurred only 6 times. However, in the simulation results of the FPGA running the algorithm, there were 24 instances where the localization error exceeded 1 m. Due to the limitations of the hardware platform, the algorithm is more prone to getting trapped in local optima during iterations. Therefore, an effective solution to this issue is required.

### 3.2. Comparative Simulation of Optimized Positioning Algorithms

In the FPGA algorithm simulation on the ModelSim platform, the simulation is set with a clock frequency of 50 MHz, the fixed-point bit width was set to 10 bits, with 30 particles and a maximum iteration count of 200. The target coordinates were set to (2, 6, 4), while the coordinates of the four nodes were (8, 2, 7), (10, 3, 1), (9, 2, 2), and (8, 1, 3). The input parameters include the positions of the four nodes and the Euclidean distances from each node to the target, assuming no measurement error (i.e., zero distance error).

#### 3.2.1. Simulation Verification of FPGA-Based VCA-PSO Localization Algorithm

**Figure 6:**
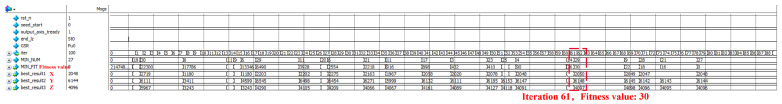
Simulating the iterative process of running the VCA-PSO positioning algorithm on FPGA.

The simulation process of the VCA-PSO localization algorithm is shown in Figure 6.

From Figure 6, it is evident that during the initial iterations, the minimum fitness value (MIN_FIT) is 22,300. As the iterations progress, there is a noticeable reduction in MIN_FIT at the 17th and 35th generations. By the 61st iteration, the MIN_FIT value drops to 30, which is close to the value achieved by the PSO algorithm at the 118th iteration, as shown in Figure 3. This indicates that the optimized localization algorithm exhibits superior convergence characteristics. By the 78th generation, the global best solution reached was (2048, 6144, 4096), which exactly matches the target coordinates (2, 6, 4).

**Figure 7:**
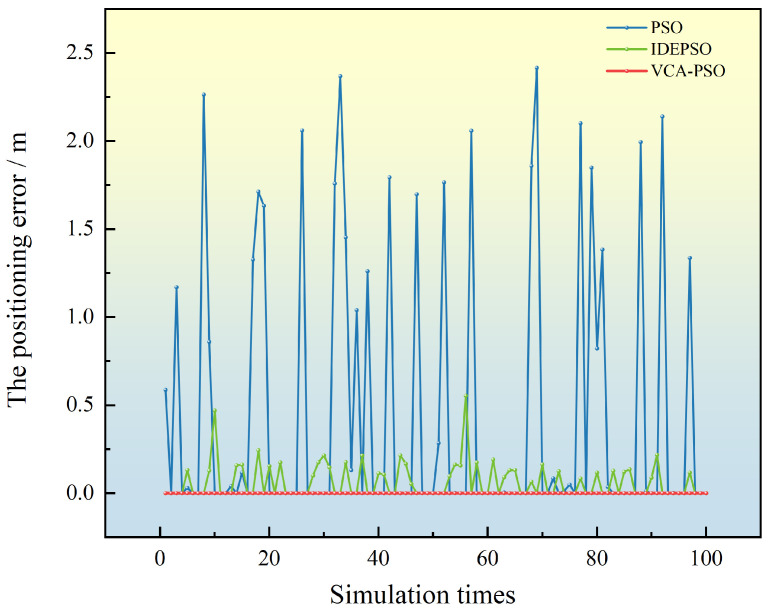
Comparison of positioning errors of the three algorithms.

If only the mutation and crossover processes of the DE algorithm are applied [29], the optimization performance on the FPGA does not achieve ideal results. A comparison between the PSO, the IDEPSO algorithm in ref [29] and VCA-PSO algorithm in terms of the localization error from 100 simulation runs is shown in Figure 7.

Although the local optimum problem of the algorithm in ref [29] has been alleviated, a comparison with the proposed VCA-PSO algorithm reveals that VCA-PSO algorithm consistently converges and completely avoids the issue of local optima, demonstrating the effectiveness of the algorithm’s optimization.

**Figure 8:**
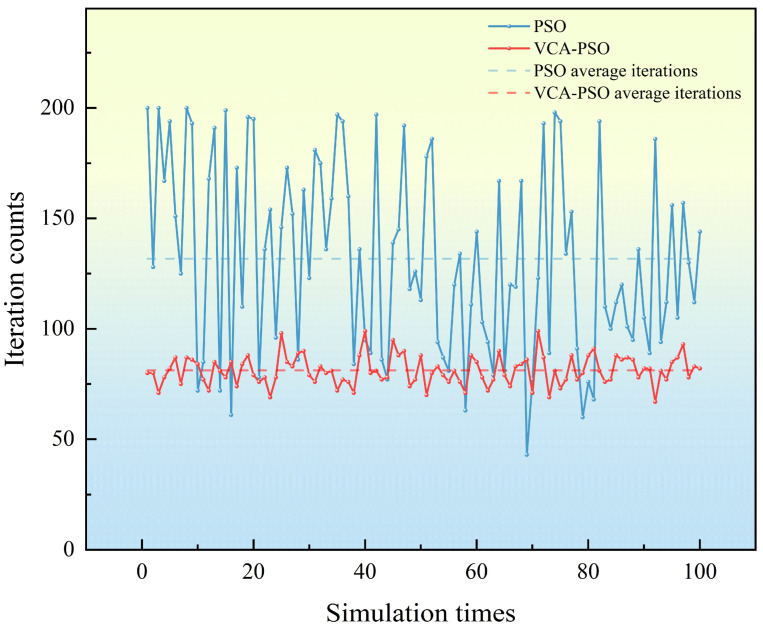
Comparison of the number of convergence of the two algorithms.

For the PSO algorithm, if convergence is not achieved within 200 iterations, the iteration count is set to the maximum value of 200 by default. The convergence comparison in Figure 8 shows that the PSO algorithm’s convergence speed is significantly slower than that of the VCA-PSO algorithm. The average iteration count for the PSO algorithm is 131.7, while the VCA-PSO algorithm achieves convergence in an average of 81.1 iterations.

Root Mean Square Error (RMSE) is commonly used as a measure of the prediction accuracy of a prediction model on continuous data, which represents the root mean square difference between the predicted and actual values, with smaller values representing a smaller degree of average deviation and higher algorithm accuracy. The RMSE of the localization algorithm is calculated as in Equation (8).

(8) $$RMSE=\sqrt{\frac{\sum_{i=1}^{n}{\left(E_{obs,i}-E_{true,i}\right)}^{2}}{n}}$$

The performance of both algorithms on the FPGA platform is presented in Table 1.

It can be found that despite the fact that the optimized algorithm includes additional operations for particle mutation, crossover, and inertia weight updates in each iteration, resulting in a 0.84-fold increase in the iteration time compared to the PSO algorithm, the overall computational time per iteration only increased by 0.13-fold due to the faster convergence speed.

#### 3.2.2. Comparative Simulation Analysis of Algorithms Under Ranging Error Conditions

The simulation results validate the feasibility, excellent localization accuracy, convergence speed, and stability of the proposed algorithm. However, since the simulation assumes ideal distance measurement conditions (i.e., zero measurement error), the localization coordinates converge precisely to the target coordinates. In practical applications, distance information obtained through methods such as TDOA or TOF, or measurements from sensors, may be influenced by hardware clock precision, environmental factors, and target characteristics, resulting in inherent measurement errors. Therefore, further simulations considering measurement errors will provide more reliable data support for evaluating the algorithm’s localization performance in real-world environments.

**Figure 9:**
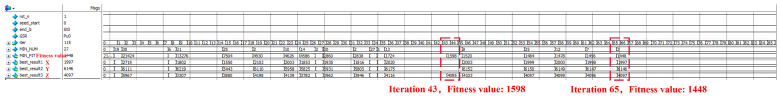
Consider the iterative process of the algorithm with a ranging error of 0.5%.

Under the condition of a 0.5% distance measurement error, the distance measurement errors from the four nodes are as follows: 0.0391 m, 0.0453 m, 0.0415 m, and 0.0394 m. The algorithm’s iteration process is shown in Figure 9.

It can be observed that the algorithm converges by the 65th iteration, with the solution being (1997, 6146, 4097), which yields an error of 0.05 m relative to the real target coordinates. During the convergence process, the MIN_FIT value drops significantly from 1598 at the 44th iteration to 1448 at convergence, with minimal fluctuation. To ensure high localization accuracy while maintaining fast solution times, a tolerance threshold for error was set during simulations with different distance measurement errors, where the algorithm is considered convergent once the localization error falls below the set threshold.

**Figure 10:**
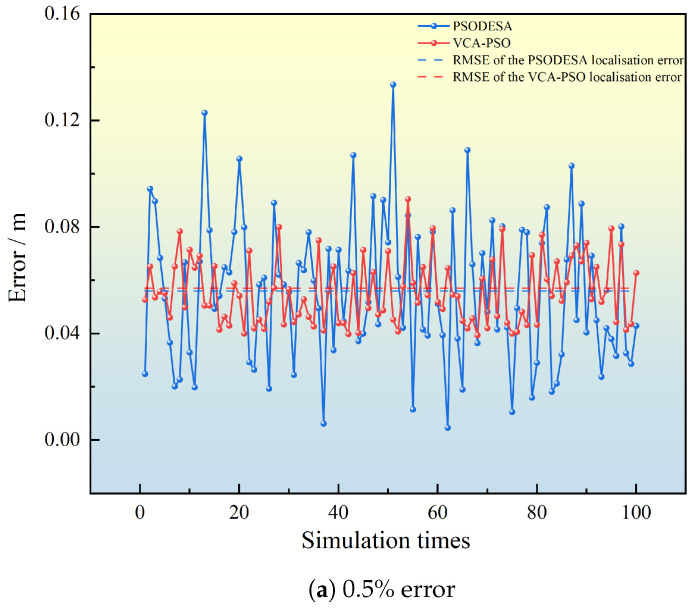

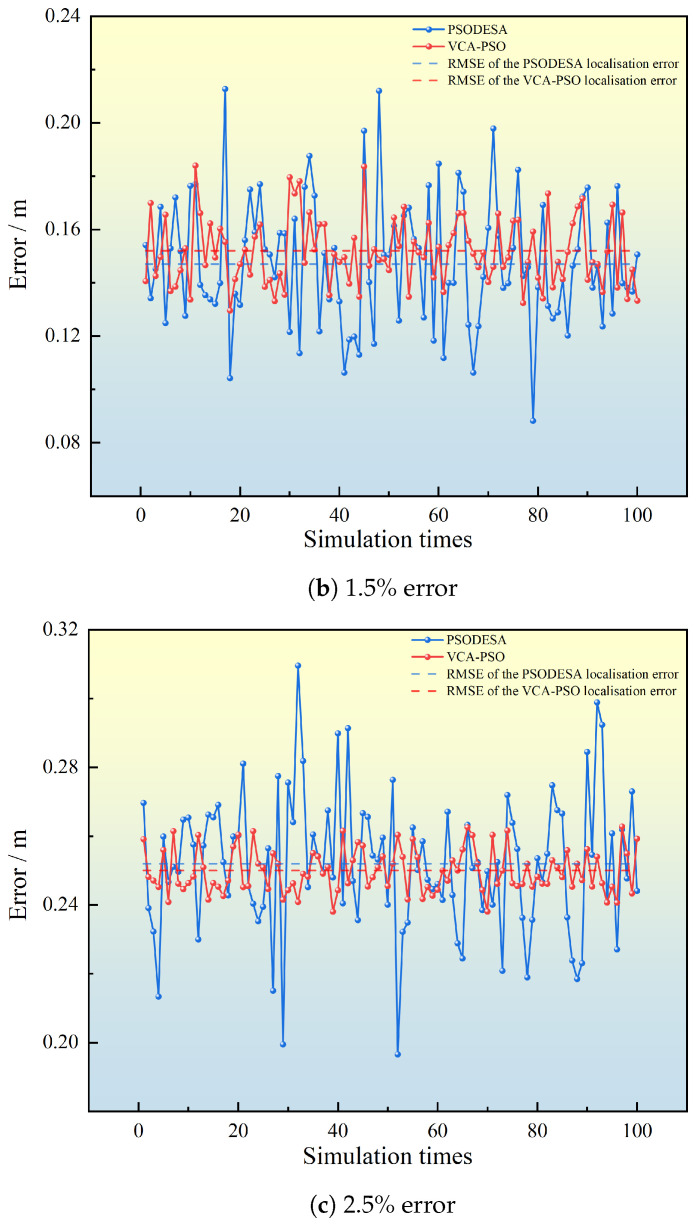
Comparative simulation of positioning errors of VCA-PSO algorithm and PSODESA algorithm with different ranging errors.

**Figure 11:**
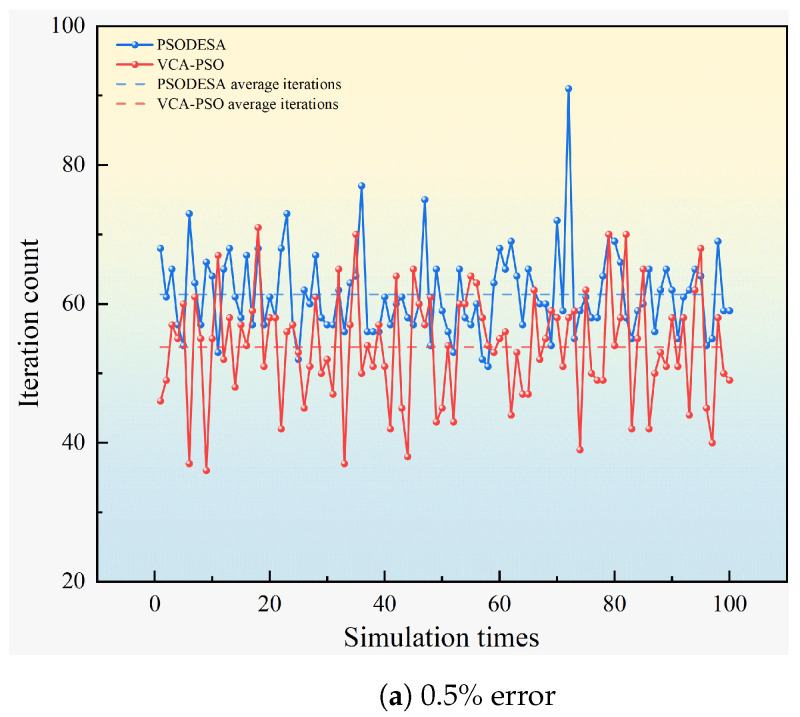

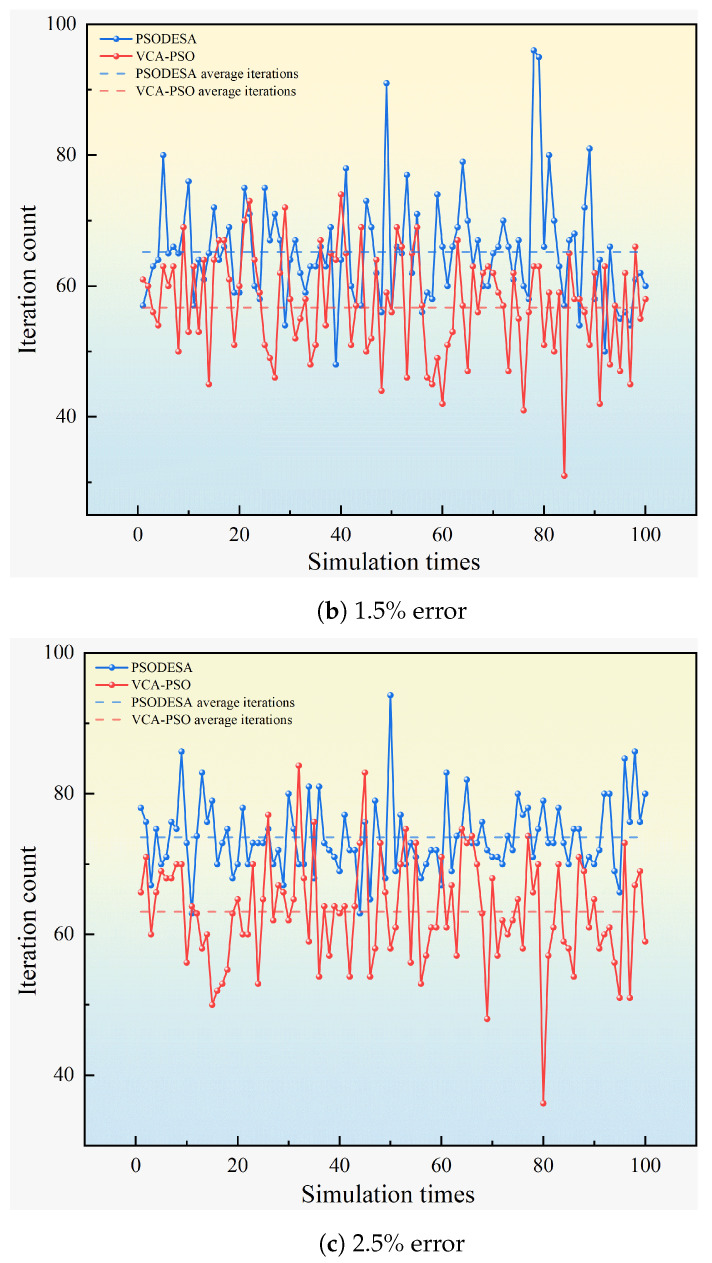
Comparative simulation of convergence times of VCA-PSO algorithm and PSODESA algorithm with different ranging errors.

In the study [30], the authors optimized the particle swarm algorithm by combining DE and SA algorithms to design the PSODESA algorithm. we applied this optimization to the collaborative positioning algorithm. Subsequently, the optimized algorithm was deployed on FPGA, and a comparative simulation was conducted with the algorithm proposed in this paper. The simulation was set up with varying ranging errors (0.5%, 1.5%, 2.5%), and through 100 simulation runs, the positioning error and convergence iterations were compared in Figure 10 and Figure 11.

The various simulation data of the algorithm for the two algorithms with different ranging errors are shown in Table 2.

The corresponding simulation data for both algorithms under different measurement error conditions are listed in the table. The comparison reveals that, in all tested measurement error scenarios, the localization errors of both algorithms are very similar. However, the algorithm in reference [30] exhibits slightly better localization accuracy than the proposed algorithm. Specifically, for measurement errors of 0.5%, 1.5%, and 2.5%, the PSODESA algorithm’s localization accuracy is higher by 0.001 m, 0.005 m, and −0.002 m, respectively.

**Figure 12:**
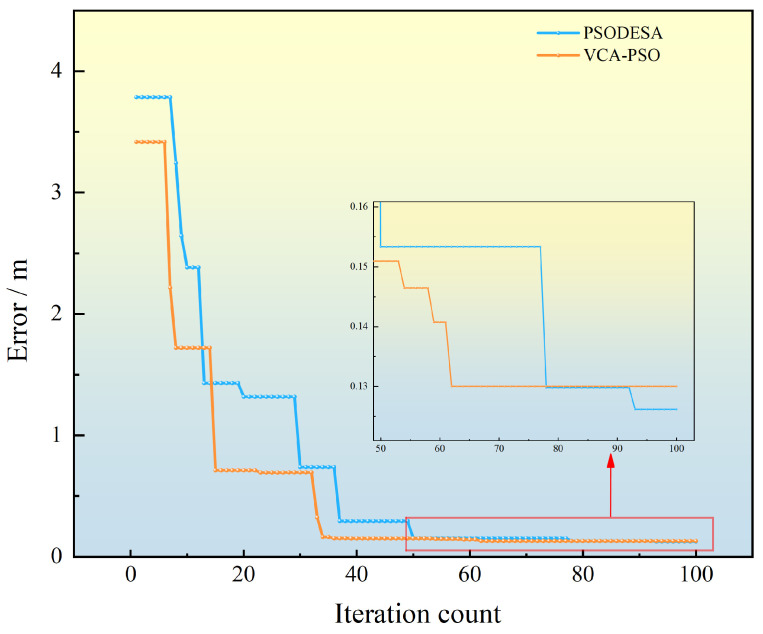
Comparison of typical convergence curves of the two algorithms.

In terms of convergence speed, the comparison of convergence curves clearly indicates that the proposed algorithm converges faster than the PSODESA algorithm. A typical convergence example is shown in Figure 12, by the time the proposed algorithm completes its computation, the PSODESA algorithm has not yet achieved the same localization accuracy and does not surpass the proposed algorithm’s precision until the 78-th iteration. Furthermore, the proposed algorithm’s convergence time remains stable across different measurement errors, maintaining convergence within 4 ms, whereas the PSODESA algorithm’s convergence time increases significantly as the measurement error increases.

Moreover, both algorithms exhibit minimal error variance, remaining within the magnitude of $10^{-4}$, confirming their high stability. Since the PSODESA algorithm is optimized based on the top-level software simulation environment, cross and mutation operation of particles throughout the iterations. Although these operations help to improve the late convergence of the algorithm, may in some cases hinder the final convergence accuracy. Specifically, these operations may introduce excessive randomness, leading the algorithm to deviate from the optimal solution during convergence, resulting in higher final localization errors and error variance compared to the proposed algorithm.

Furthermore, based on the XC6SLX16-2FTG256 chip, a comparison of the resource consumption for the three algorithms after deployment is presented, as shown in Table 3.

It can be observed that the optimized algorithm consumes noticeably more resources than the basic PSO algorithm. However, the resource consumption of the PSODESA algorithm is very similar to that of the VCA-PSO algorithm proposed in this paper, and both exhibit an extremely low consumption rate relative to the total hardware resources available on the chip. While the PSODESA algorithm marginally outperforms the proposed algorithm in terms of accuracy, the proposed algorithm demonstrates a distinct advantage in terms of convergence speed and stability. This makes the proposed algorithm more promising for practical applications, particularly in real-time scenarios where rapid computation is crucial. Thus, the effectiveness of the optimizations made in this paper is demonstrated in the FPGA computing architecture.

#### 3.2.3. Impact of Particle Count on FPGA Resource Consumption

An analysis was conducted on the variation in FPGA resource consumption under different particle numbers. Based on the xc6slx16-2ftg256 chip, resource consumption was recorded for particle numbers increased to 60 and 90, both after synthesis and implement design, according to the simulation report. Additionally, the impact on single-iteration time and convergence iterations was compared, as shown in Table 4. For each particle number, the first row represents resource consumption after synthesis, while the second row represents resource consumption after implement design.

It can be observed that despite the exponential increase in the number of particles, the resource consumption after implement design does not show a significant difference. Moreover, relative to the total chip resources, the algorithm maintains an extremely low resource utilization rate. However, ModelSim simulations reveal that the time required for a single iteration increases proportionally, which aligns with the time complexity analysis in Section 2.4. Additionally, although the number of convergence iterations decreases as the number of particles increases, the relationship is not strictly linear. This conclusion provides valuable insights for selecting appropriate algorithm parameters in practical engineering applications.

#### 3.2.4. Analysis of the Relationship Between Ranging Error and Localization Error in the VCA-PSO Algorithm

For the fitness function (3), the input error originates from the distance measurement $\Delta d_{i}$. We define $r_{i}=\sqrt{{\left(x-x_{si}\right)}^{2}+{\left(y-y_{si}\right)}^{2}+{\left(z-z_{si}\right)}^{2}}$, and the one-way error propagation is represented as $f_{i}\left(X\right)=\left|r_{i}-\Delta d_{i}\right|$. By taking the partial derivative with respect to $\Delta d_{i}$, we can obtain Equation (9).

(9) $$\frac{\partial f_{i}}{\partial d_{i}}=\left\{\begin{array}{c} -1,r_{i}-d_{i}>0\\ 1,r_{i}-d_{i}<0 \end{array}\right.$$

Aggregating the error propagation from all $\Delta d_{i}$, the output error is denoted as Δf.

(10) $$\Delta f=\sqrt{\sum_{i=1}^{n}{\left(\frac{\partial f_{i}}{\partial d_{i}}\Delta d_{i}\right)}^{2}}$$

Consequently, the relationship between RMSE and the error can be approximated by Equation (11).

(11) $$RMSE=k\sqrt{\sum_{i=1}^{n}{\left(\Delta d_{i}\right)}^{2}}+c$$

Based on 33 simulation trials, the final solution yields k=0.5091 and c=0.0231, leading to Equation (12).

(12) $$RMSE=0.5091\sqrt{\sum_{i=1}^{n}{\left(\Delta d_{i}\right)}^{2}}+0.0231$$

**Figure 13:**
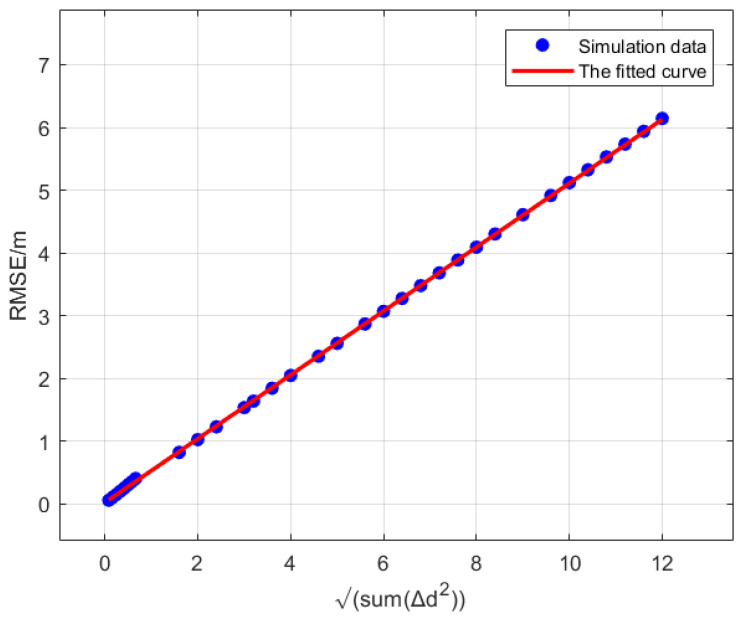
The RMSE error curve.

The RMSE error curve is shown in Figure 13.

## 4. Four-Node Static Co-Positioning Experiment

**Figure 14:**
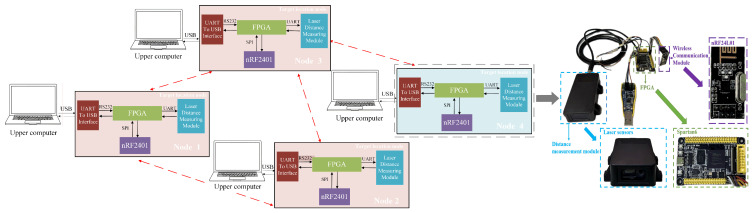
Schematic diagram of the overall structure and positioning nodes of the co-location system.

To validate the algorithm’s performance under real-world conditions, we conducted a multi-node collaborative target localization experiment. A distributed network structure was chosen as the communication topology for the multi-node system, and a four-node collaborative positioning platform was built based on xc6slx16-2ftg256 chip of FPGA, nRF2401 module, and laser sensors. By default, each node knows its own coordinates. Node 1 is the master node, responsible for receiving ranging and positioning information from all other nodes and transmitting the computed target coordinates to each node. After receiving the target coordinates, each node uploads them to the host computer. The overall structure and individual node layout are shown in Figure 14.

Where nodes 1, 2, and 3 form a group, with bidirectional communication between each pair of nodes. Node 4 is part of another group, and nodes 1 and 2 cannot directly communicate with node 4; they rely on node 3 to forward data.

### 4.1. Collaborative Localization Experiment for Stationary Targets

**Figure 15:**
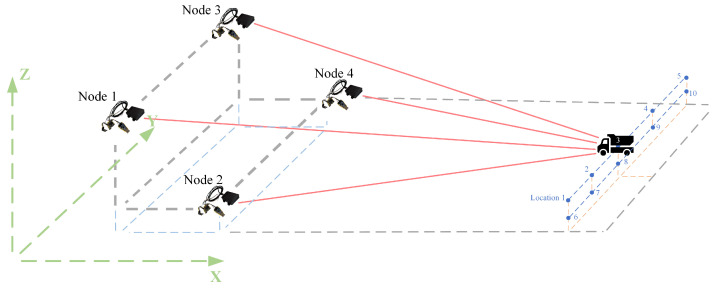
Schematic diagram of the test scene.

The experimental layout is shown in Figure 15.

**Figure 16:**
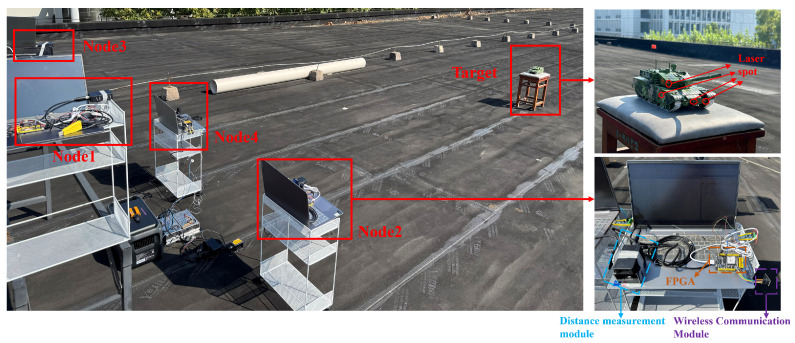
Positioning test site.

The four nodes are placed at different heights and positions on customized racks, with coordinates of (1, 1, 1), (2, 1, 0.5), (1, 3, 1), and (2, 3, 0.5). The target is placed randomly at ten different positions: (5, 1.2, 0.5), (5, 1.6, 0.5), (5, 2, 0.5), (5, 2.4, 0.5), (5, 2.8, 0.5), (5, 1.2, 1), (5, 1.6, 1), (5, 2, 1), (5, 2.4, 1), and (5, 3, 1). The test site is shown in Figure 16.

**Figure 17:**
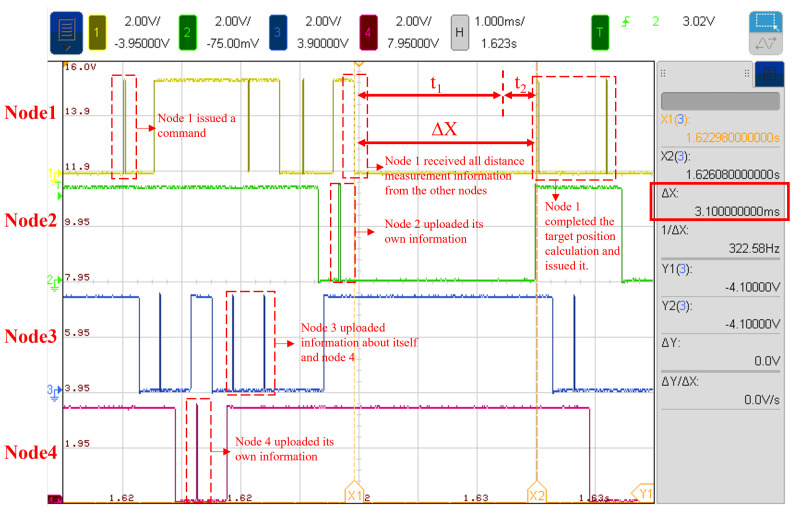
Oscilloscope−measured changes in radio transceiver control signals at each node.

When the target is located at (5, 2, 0.5), the changes in the send/receive control signals for each node and the collaborative localization results are shown in Figure 17.

By monitoring the send/receive control signals (RF_CE signal) with an oscilloscope, it is confirmed that the nodes are correctly performing the data transmission and reception during the collaborative localization process, according to the designed strategy. The waveform of node 1’s transmission control indicates that after node 1 receives the ranging and positioning information from the other nodes, it begins to issue the target coordinates for solving. The oscilloscope measured the total time, ΔX, as 3.1 ms, where ΔX is the sum of the algorithm’s solving time ($t_{1}$) and the communication chip configuration time ($t_{2}$). Therefore, the conclusion is that the time required by node 1 to solve the target coordinates ($t_{1}$) does not exceed 3.1 ms, which is consistent with the simulation results, validating the high efficiency of FPGA in the positioning algorithm.

**Figure 18:**
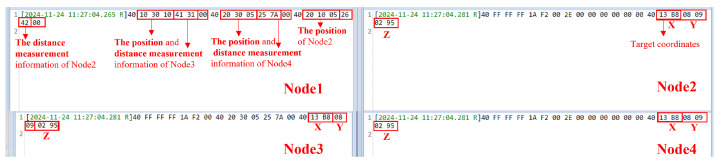
Information on data uploaded by nodes.

In Figure 18, the data ‘FF FF FF 1A F2 00’ represents the command issued by node 1 to instruct the remaining nodes to upload their ranging data. It can be observed that node 1 sequentially receives the position and ranging information from nodes 3, 4, and 2. In this data stream, the first byte, 40, indicates that the data was received normally; the following three bytes represent the node’s position information along the X, Y, and Z axes, and the subsequent two bytes correspond to the ranging information.

In the data uploaded by each node, the starting byte of 40 indicates that the reception status is normal, and 2E indicates that the transmission is normal. Based on the measurement results, the ranging errors for node 1, node 2, node 3, and node 4 are 0.1578 m (error: 3.8%), 0.07 m (error: 2.2%), 0.1163 m (error: 2.8%), and 0.1012 m (error: 3.1%), respectively. The final 6 bytes received by nodes 2, 3, and 4 represent the predicted target location for this measurement, with the X, Y, and Z coordinates each encoded in two bytes. By using the final uploaded results from nodes 2, 3, and 4, the solved target coordinates are (13B8, 0809, 0295), which, when converted to decimal, are (5048, 2057, 661). The positioning error is 0.1618 m.

The data collected by the host computer confirms that the four nodes can successfully perform distributed communication and collaborative localization.

**Figure 19:**
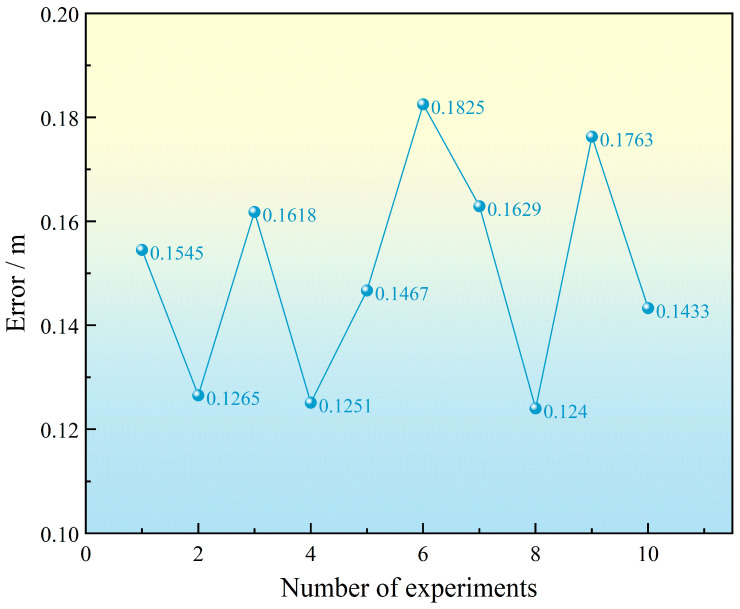
Positioning results from ten trials.

The results of the positioning errors obtained from ten positioning experiments are shown in Figure 19.

The RMSE for ten trials is approximately 0.152 m. Due to node position deviations, changes in outdoor lighting, target shape, and other environmental factors, the ranging results exhibit some random errors, causing slight discrepancies between the actual positioning results and the simulation results. However, the positioning error variance remains on the order of $10^{-4}$, with a value of 0.00044, indicating that the algorithm demonstrates extremely high stability.

### 4.2. Cooperative Localization Experiment Under Target Motion Conditions

To achieve continuous collaborative localization of a moving target, the algorithm’s scalability for target tracking can be validated. Two factors must be considered in the experiment. First, all nodes must acquire distance measurement data simultaneously; second, the sampling frequency should be matched to the target’s speed to obtain observable results. According to the oscilloscope waveform in Figure 17, the four nodes complete one collaborative localization within 10 ms. However, due to the relatively slow target speed in the experiment, a higher sampling frequency does not yield satisfactory observable results, and thus thesampling frequency is set to 5 Hz.

To ensure that all four nodes obtain distance measurement information concurrently, a time synchronization strategy is implemented, where the unidirectional wireless transmission time $T_{1}$ between two adjacent nodes is determined by Equation (13).

(13) $$T_{1}=t_{\mathrm{spi}}+t_{\mathrm{pack}}+t_{\mathrm{emit}}+t_{\mathrm{transmission}}$$

where the spi transmission time $t_{\mathrm{spi}}$ is determined by the clock frequency specified in the transmission protocol; the RF chip’s data packaging time $t_{\mathrm{pack}}$ depends on the volume of data; the time required for data emission via the antenna, $t_{\mathrm{emit}}$, is determined by the inherent speed of the chip; and since data propagates as electromagnetic waves through the air, the air transmission time $t_{\mathrm{transmission}}$ is negligible. Under the designed distributed communication framework, the data transmission delay *T* between nodes is given by Equation (14).

(14) $$T=T_{1}+n\left(T_{1}+T_{\mathrm{soft}}\right)+\left(m-1\right)T_{\mathrm{delay}}$$

where $T_{\mathrm{soft}}$ represents the data processing and delay time on the FPGA; $T_{\mathrm{delay}}$ denotes the interval between successive command transmissions from node 1; *n* is the number of hops that the data traverses; and *m* is the number of times node 1 issues a request for ranging data.

According to Equation (14), if node 1 initiates the next sensor data acquisition after a transmission time *t*, the other nodes will adaptively delay by (t−T).

The triggering condition for the initial sampling of all nodes is that when the target, at its preset starting position, moves sufficiently to break the connection between two wires, all four nodes are simultaneously triggered to perform their first sensor data acquisition.

**Figure 20:**
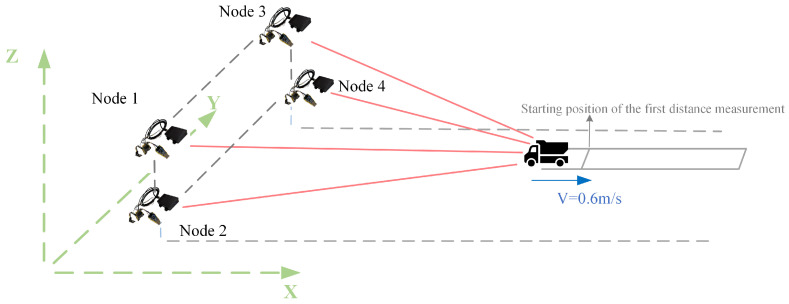
Schematic diagram of the test scene.

The experimental layout is shown in Figure 20.

**Figure 21:**
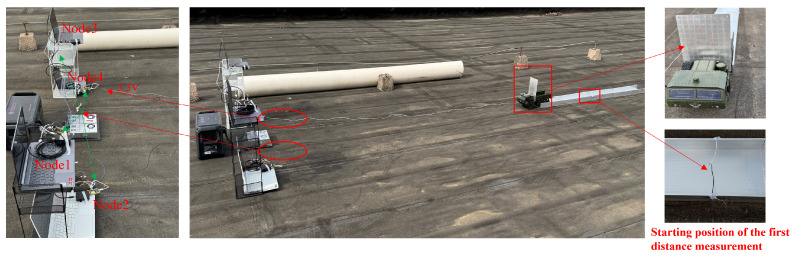
Positioning test site.

The nodes are positioned at (1, 1.4, 1.42), (1, 1.4, 1.04), (1, 2.6, 1.42), and (1, 2.6, 1.04), respectively. The target moves along a designated trajectory at a constant speed of 0.6 m/s. When the target reaches (5, 2, 1.18), the four nodes commence collaborative localization, as depicted in the accompanying schematic. The test site is shown in Figure 21.

**Figure 22:**
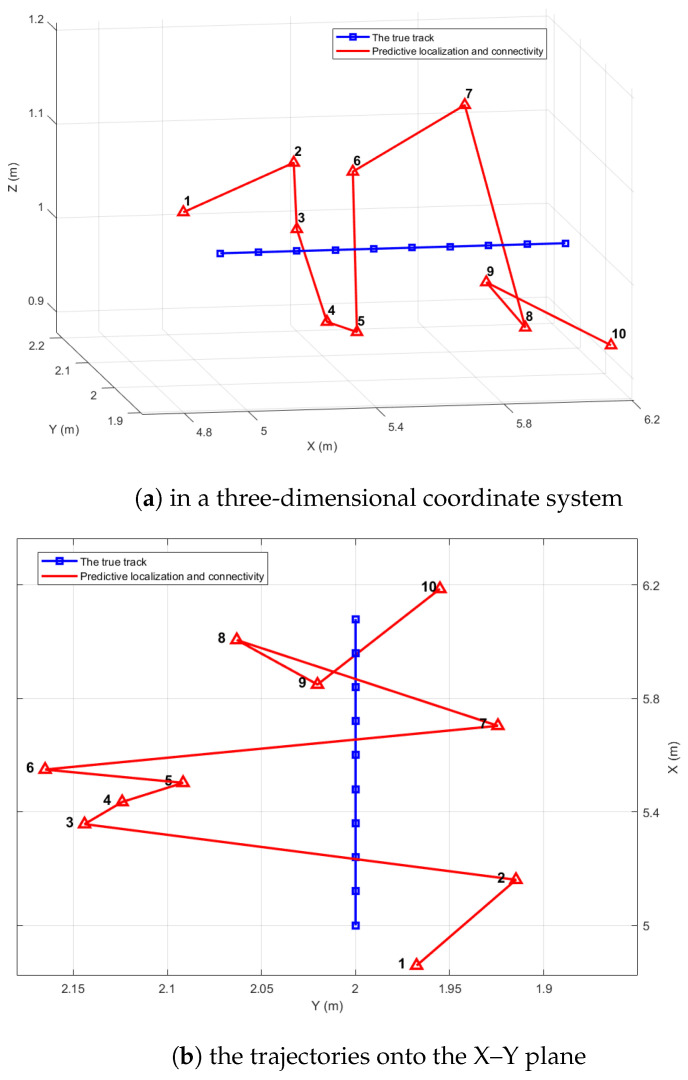
Comparison between the continuously predicted positions and the target trajectory.

The final comparison between the continuously predicted positions from ten consecutive iterations and the target trajectory is illustrated in Figure 22. Figure 22a presents the target trajectory alongside the predicted positions in a three-dimensional coordinate system, while Figure 22b shows the projection of the trajectories onto the X–Y plane.

Figure 22 demonstrates that, with an appropriately configured sampling frequency, the predicted positions from the four-node cooperative localization system exhibit a clear trend of moving along with the target trajectory.

**Figure 23:**
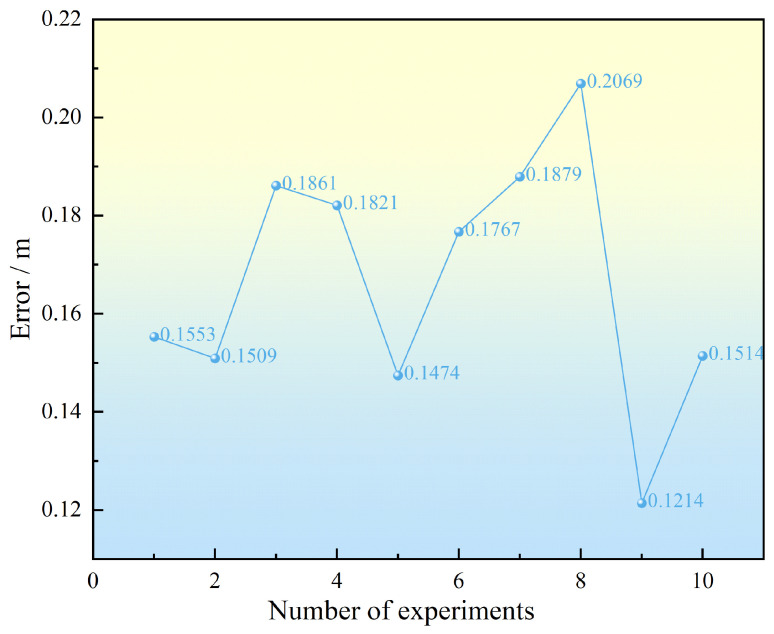
Positioning results from ten trials.

The error for the ten localization results is shown in Figure 23.

Based on the experimental analysis, the localization error has an RMSE of 0.1683 m and a variance of 0.0007. The experimental results confirm that the proposed algorithm exhibits stable localization and reliable performance in tracking moving targets. Moreover, the designed four-node cooperative localization algorithm achieves a solution frequency of up to 100 Hz, which meets the real-time requirements for target tracking. Therefore, this algorithm can provide dependable observational input for engineering applications in moving target tracking.

## 5. Conclusions

In addressing the issue of precise target detection in collaborative operations of unmanned swarms, this paper designs a particle swarm localization algorithm based on FPGA and implements rapid iterative convergence of the algorithm through four modules. Considering that FPGA-based particle swarm algorithm are prone to falling into local optima, this paper introduces particle mutation, variable crossover probability functions, and variable inertia weight functions to ensure the reliable convergence of the algorithm.

Through comparative simulations with algorithm from other literature under different ranging error conditions, the results show that the proposed algorithm maintains high localization accuracy while demonstrating faster solution speeds and greater stability. Furthermore, localization test through cooperative communication of four nodes validates the algorithm’s reliability, speed, and stability in practical localization experiments.

## Figures and Tables

**Table 1:** Comparison of the positioning performance of the two algorithms.

Type of Algorithm	RMSE/m	Single Iteration Time/ms	Average Convergence Time/ms
PSO	0.8733	0.031	4.08
VCA-PSO	0	0.057	4.6

**Table 2:** Specific parameters of the performance of the two algorithms with different ranging errors.

Ranging Errors	VCA-PSO				PSODESA			
	RMSE/m	Variance/m^2^	Average Number of Iterations	Average Convergence Time/ms	RMSE/m	Variance/m^2^	Average Number of Iterations	Average Convergence Time/ms
0.5%	0.057	$1.04\times 10^{-4}$	53.7	3.06	0.056	$7.3\times 10^{-4}$	61.3	3.49
1.5%	0.152	$1.58\times 10^{-4}$	56.7	3.23	0.147	$6.5\times 10^{-4}$	65.5	3.73
2.5%	0.25	$3.91\times 10^{-5}$	63.2	3.6	0.252	$4\times 10^{-4}$	72.8	4.15

**Table 3:** Comparison table of resource consumption of various algorithms after implement design.

Type of Algorithm	Slice Register	Slice Luts	Lut-ff Pairs	RAM/FIFO	DSP48A1s
PSO	210	411	219	3	1
PSODESA in ref [30]	260	445	226	5	2
VCA-PSO	263	458	232	4	1

**Table 4:** Table of FPGA resource consumption for different particle numbers.

Number of Particles	Slice-Register	Slice-Luts	Lut-ff Pairs	RAM/FIFO	DSP48A1s	Single Iteration Time/ms	Average Convergence
30	3033	4658	2579	10	19	0.057	81.1
	263	458	232	4	1		
60	4595	6631	4111	10	19	0.115	75.8
	263	487	236	4	1		
90	6125	8171	5405	10	19	0.173	73.3
	269	490	240	4	1		

## Data Availability

The original contributions presented in the study are included in the article, further inquiries can be directed to the corresponding authors.
